# Incidental Splenic Leiomyoma in a Child Uncovered During Workup for Eosinophilic Gastroenteritis

**DOI:** 10.7759/cureus.94027

**Published:** 2025-10-07

**Authors:** Reza Khorvash, Maryam Monajemzadeh

**Affiliations:** 1 Osteopathic Medicine, Midwestern University Arizona College of Osteopathic Medicine, Glendale, USA; 2 Pathology and Laboratory Medicine, McMaster University, Hamilton, CAN

**Keywords:** eosinophilic gastroenteritis, leiomyoma, pediatric, spleen, splenectomy

## Abstract

Eosinophilic gastroenteritis (EGE) is a rare, heterogeneous inflammatory disorder characterized by eosinophilic infiltration of the gastrointestinal tract in the absence of secondary causes. Clinical presentation is variable and often mimics other gastrointestinal conditions, making diagnosis challenging. Splenic leiomyoma, in contrast, is a rare benign smooth muscle tumor, typically reported in immunocompromised patients. Pediatric cases are exceedingly uncommon.

We describe a 9-year-old boy presenting with persistent diarrhea and peripheral blood eosinophilia. Endoscopy showed esophageal furrowing, gastric and colonic erythema, and biopsies demonstrated marked eosinophilic infiltration in multiple gastrointestinal sites, consistent with EGE. During work-up, an abdominal ultrasound identified a well-defined splenic mass. Splenectomy revealed a solitary spindle cell tumor composed of bland smooth muscle fibers, diffusely positive for desmin and smooth muscle actin, and negative for C-Kit, CD34, and S100, confirming the diagnosis of splenic leiomyoma.

The coexistence of EGE and splenic leiomyoma in an immunocompetent pediatric patient is highly unusual. While the splenic lesion may represent an incidental finding, its occurrence in a child with an inflammatory gastrointestinal disorder raises the possibility of broader clinical contexts for splenic leiomyoma beyond immunodeficiency. This case expands the clinical spectrum of splenic leiomyoma and underscores the importance of thorough investigation in children with persistent gastrointestinal symptoms. We present the first reported case of EGE associated with an incidental splenic leiomyoma in an immunocompetent child. Documentation of such cases contributes to understanding the spectrum, pathogenesis, and potential associations of this rare tumor.

## Introduction

Eosinophilic gastroenteritis (EGE) is an uncommon, heterogeneous inflammatory disorder of the gastrointestinal tract characterized by eosinophilic infiltration in the absence of causes such as parasitic infections, drug reactions, or systemic diseases [1,2]. The clinical manifestations are diverse and largely depend on the distribution of eosinophilic infiltration across the gastrointestinal tract. Patients may present with nonspecific symptoms, including abdominal pain, diarrhea, vomiting, nausea, gastrointestinal bleeding, protein-losing enteropathy, and, in severe cases, malnutrition [3,4]. Because eosinophils are generally present in the gastrointestinal tract, the diagnosis requires clinicopathologic correlation, recognition of abnormal eosinophil density, and exclusion of secondary causes [5]. Diagnosis of EGE is particularly challenging, often mimicking inflammatory bowel disease, infectious enterocolitis, or food allergy [6]. Its pathogenesis is incompletely understood but appears to be multifactorial. Immune dysregulation with a Th2-mediated response, hypersensitivity to allergens, and genetic predisposition have all been implicated [7,8]. Increased levels of cytokines, such as IL-5, and chemokines, such as eotaxin, recruit and activate eosinophils, leading to mucosal injury and chronic inflammation [4]. Endoscopic findings range from normal mucosa to nonspecific erythema, friability, or ulcers [9]. Treatment usually involves corticosteroids, dietary modification, and, in refractory cases, biologic agents [10].

Splenic leiomyoma, on the other hand, is a rare benign smooth muscle tumor, usually arising from the splenic capsule, trabeculae, or vascular structures. Few cases have been reported in the literature, and most were identified incidentally or in patients with underlying immunodeficiency such as HIV/AIDS, post-transplant immunosuppression, or ataxia-telangiectasia [11-13]. In the pediatric population, benign splenic tumors are themselves rare, with cysts, hamartomas, and hemangiomas being more common, while leiomyomas are extraordinarily unusual [14]. Susmitha et al. also highlighted that splenic leiomyomas may present asymptomatically, often discovered during evaluation for unrelated conditions [12].

We report the case of a 9-year-old boy with EGE who presented with diarrhea. During the investigation, an incidental solitary splenic mass was identified and histologically confirmed as a leiomyoma. The first published pediatric case of splenic leiomyoma involved a boy with ataxia-telangiectasia, further emphasizing its exceptional rarity [15]. In a retrospective review of 30 pediatric cases with benign splenic lesions, leiomyoma was identified in only one patient, underscoring its rarity [16].

## Case presentation

A 9-year-old boy was referred for evaluation of persistent diarrhea. His past medical history was unremarkable, with no evidence of chronic illness, acute infections, or prior gastrointestinal disorders. Routine laboratory investigations, including complete blood count and biochemical profile, were primarily within normal limits, except for peripheral blood eosinophilia. Stool cultures and parasitological examinations showed no significant findings. Given the persistence of symptoms, endoscopic evaluation and abdominal ultrasound were performed.

**Figure 1:**
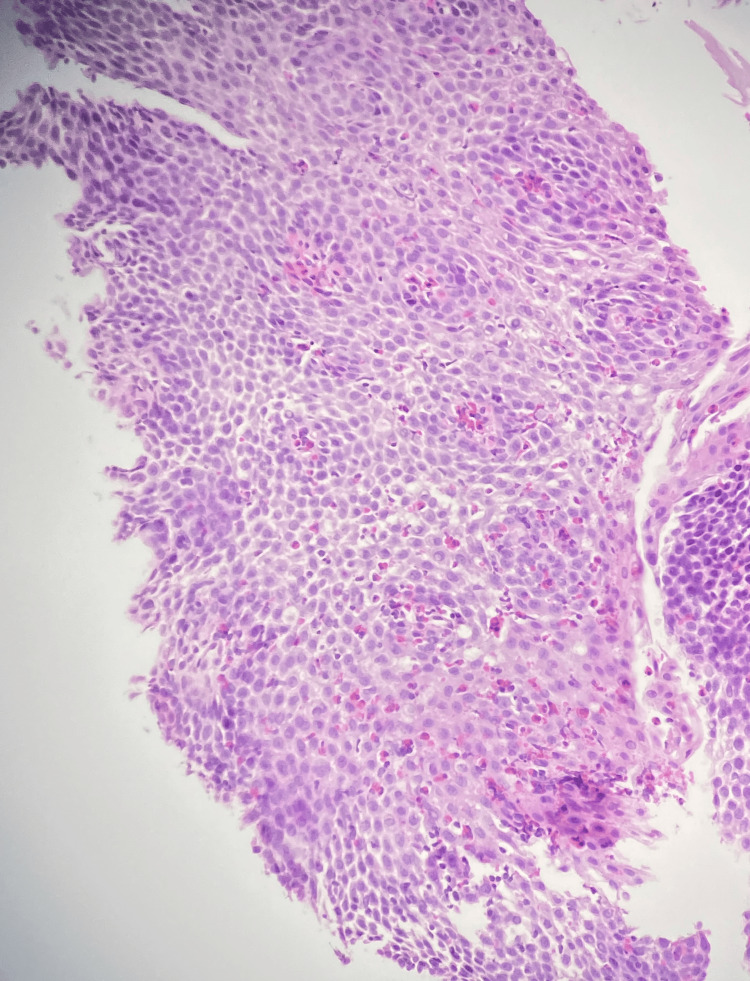
Eosinophils infiltrating the esophageal squamous mucosa

**Figure 2:**
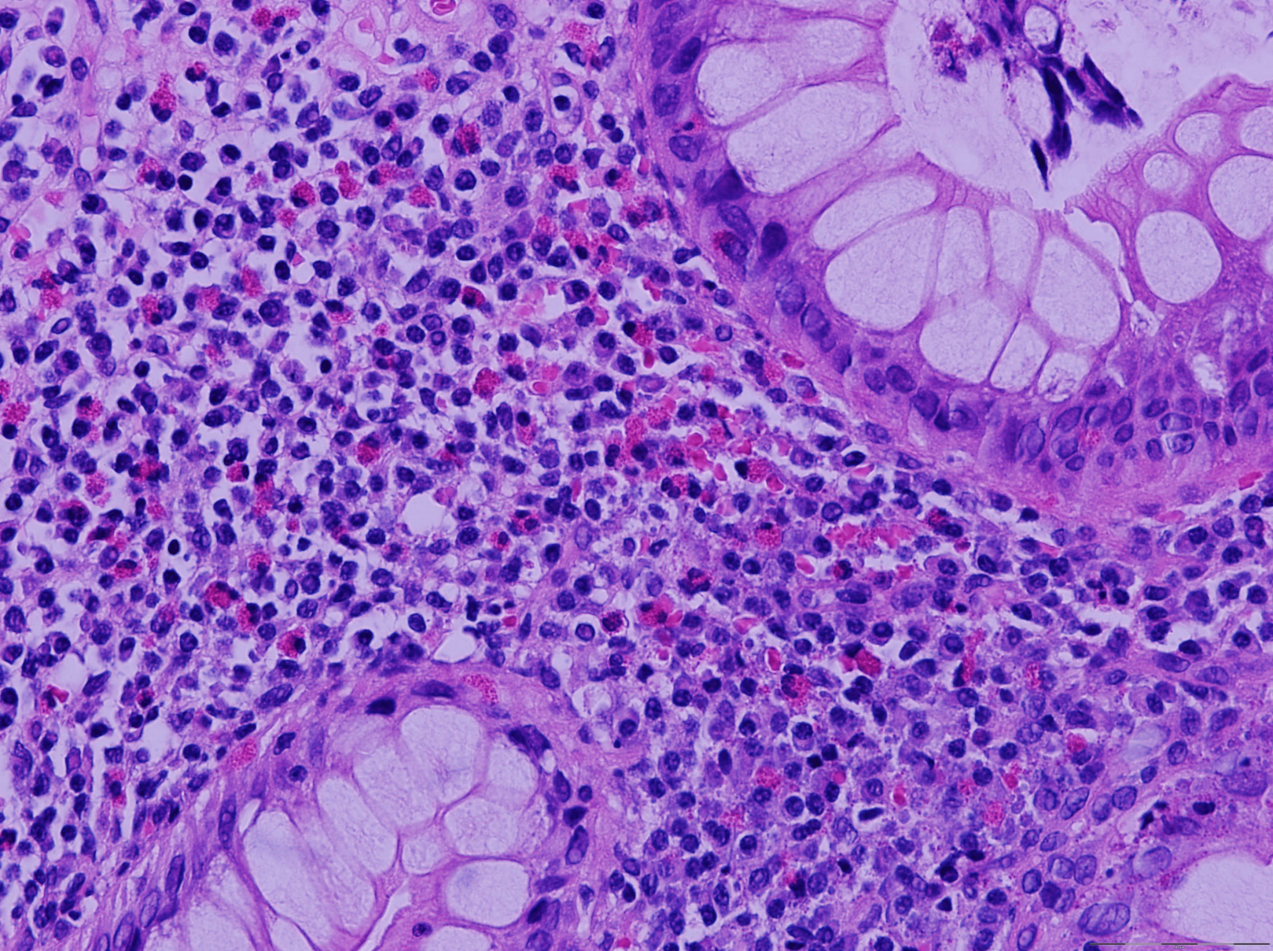
Large intestinal mucosa with eosinophil infiltration

Upper gastrointestinal endoscopy and colonoscopy revealed esophageal furrowing and trachealization, as well as gastric and colonic erythema, without evidence of ulcers, polyps, or other discrete lesions. Multiple biopsies were obtained from the esophagus, stomach, duodenum, and colon. Histopathologic examination demonstrated marked eosinophilic predominant infiltration in all samples, including dense mucosal eosinophilia, more than 50 high-power fields or HPF, epithelial injury as eosinophilic pititis and cryptitis in the gastric and colon, respectively, and degranulation, suggestive of EGE. Figures 1, 2 show esophageal and duodenal biopsies infiltrated with eosinophils, respectively. 

**Figure 3:**
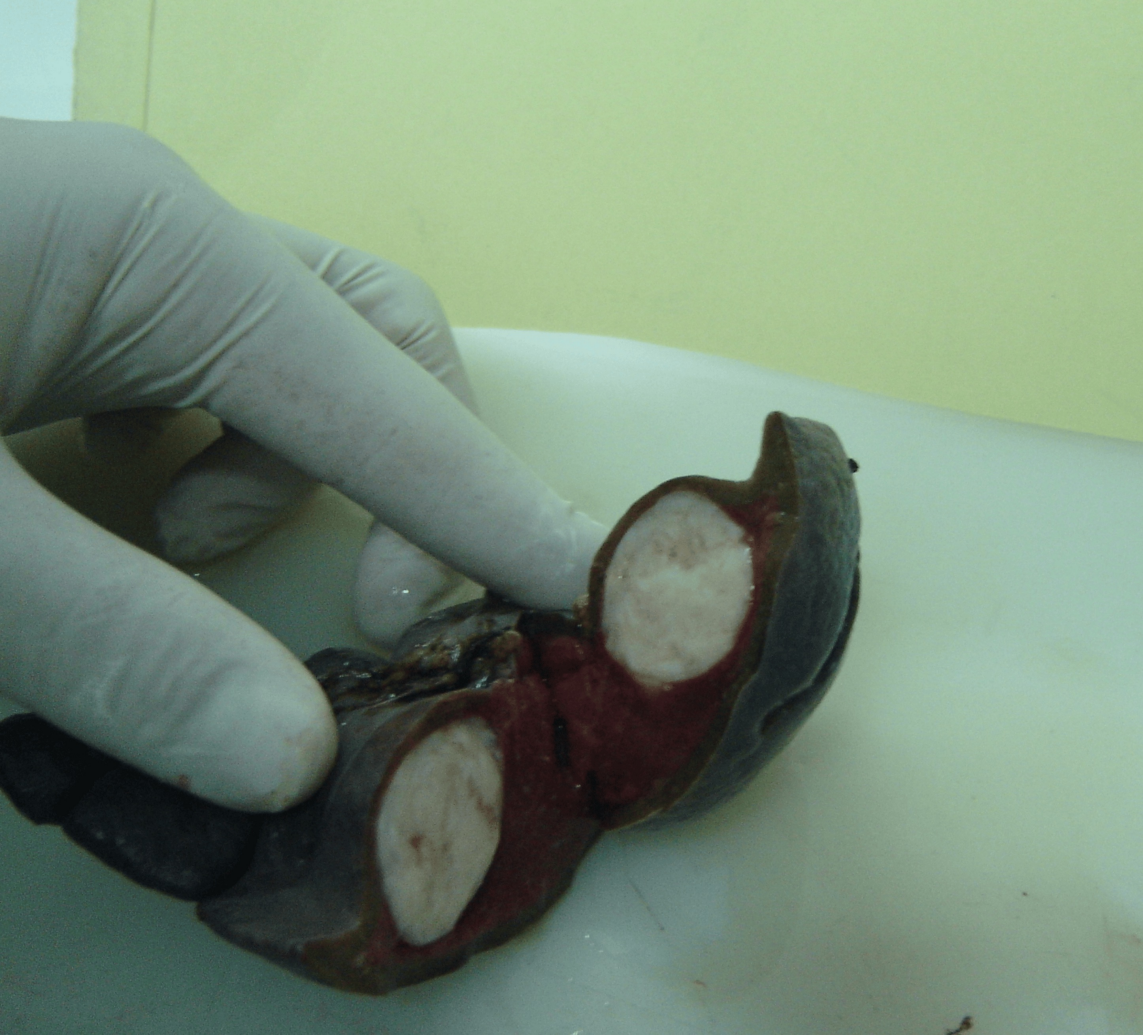
Well-demarcated splenic mass

**Figure 4:**
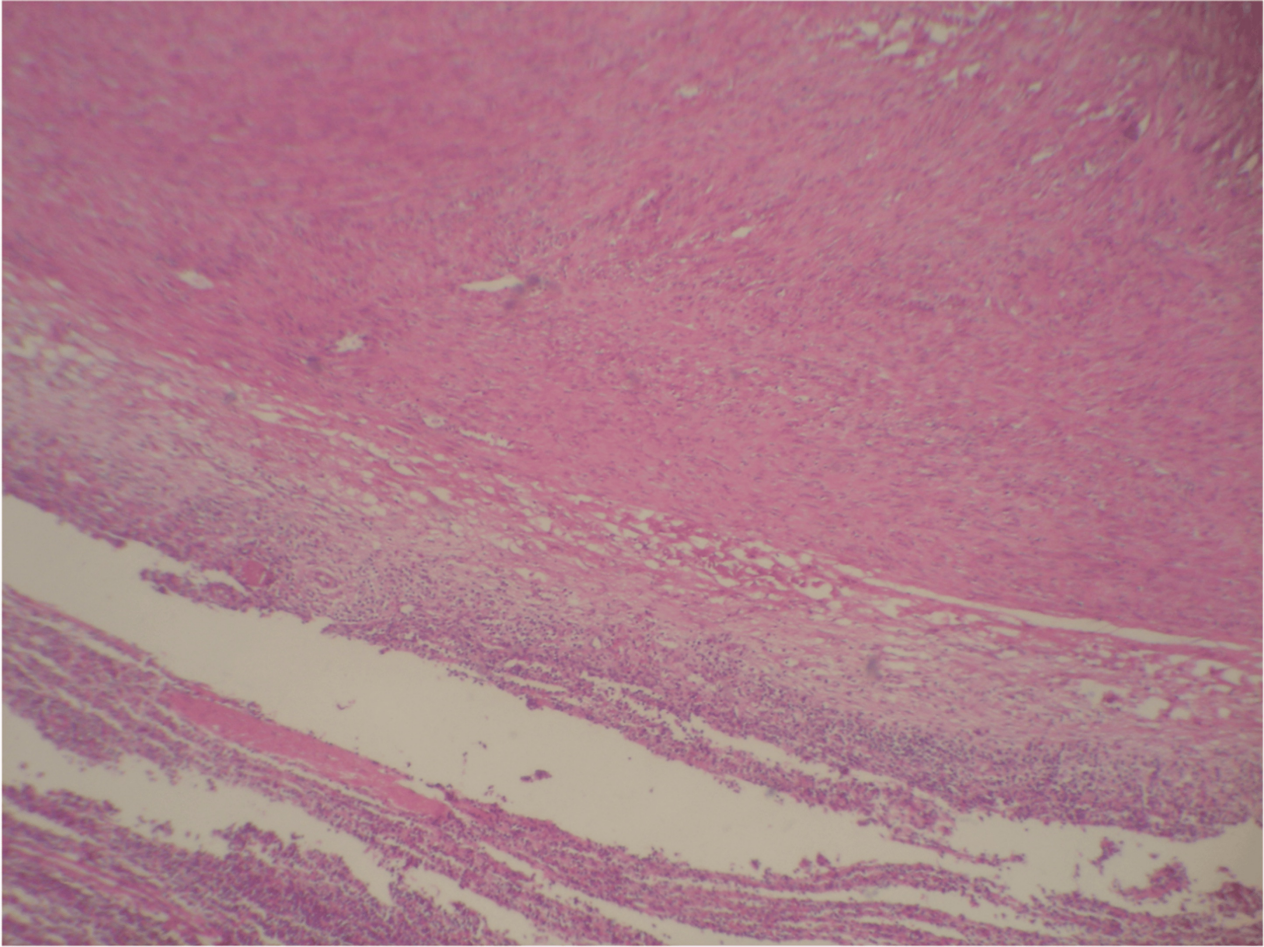
Spleen with the leiomyoma

**Figure 5:**
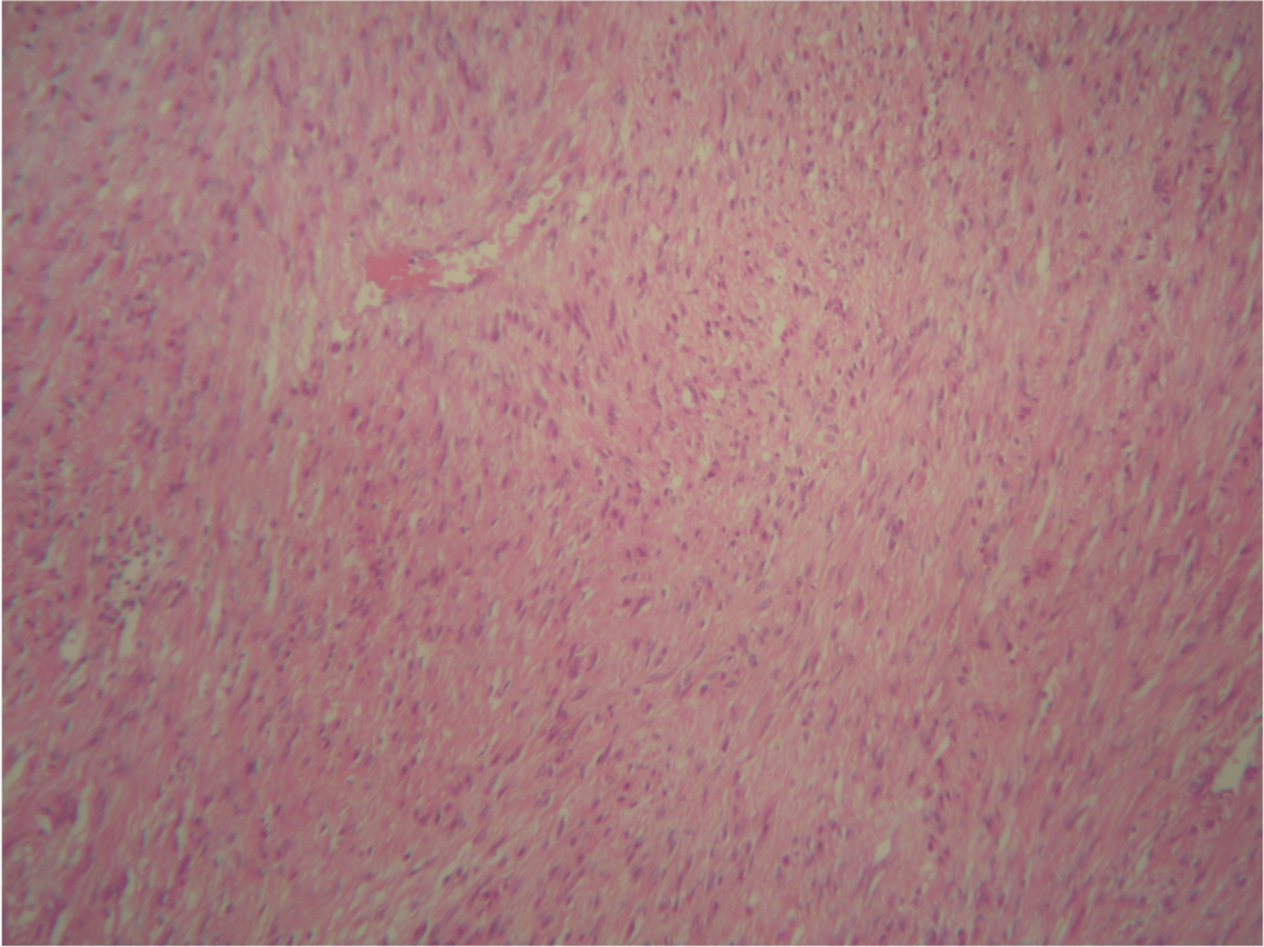
High-power view shows bland-looking spindle cell tumor cells

Abdominal ultrasonography revealed a well-defined mass in the spleen. The differential diagnosis for this mass included abscess, hamartoma, and neoplastic processes, such as lymphoma. Due to the uncertain nature of the lesion, a surgical excision was performed. Gross examination of the splenectomy specimen showed a solitary, well-circumscribed intraparenchymal mass (Figure 3). Histopathological evaluation showed a spindle cell neoplasm composed of bland-looking smooth muscle fibers arranged in intersecting fascicles, without atypia, necrosis, or significant mitotic activity. Immunohistochemistry confirmed the smooth muscle phenotype, with tumor cells diffusely positive for desmin and smooth muscle actin (Figures 4, 5) and negative for C-Kit, CD34, and S100. Congo red staining demonstrated no amyloid deposition. These findings established the diagnosis of a benign smooth muscle tumor consistent with splenic leiomyoma.

Follow-up after 6 months showed significant symptomatic improvement with dietary modification and proton pump inhibitor therapy. Repeat endoscopy demonstrated mild eosinophilic infiltration within the esophagus, duodenum, and colon.

## Discussion

Due to its heterogeneous presentation and overlap with other gastrointestinal conditions, EGE is a diagnostic challenge. In this patient, the presence of diffuse eosinophilic infiltration within the esophagus, stomach, duodenum, and colon highlighted the widespread nature of the disease. The finding of massive esophageal and eosinophilic cryptitis and pititis underscores the mucosal injury, which is consistent with EGE [1,17,18]. Notably, there were no other causes, such as parasitic infections, drug reactions, or systemic disorders. 

The accidental finding of a splenic leiomyoma during the examination is significant. Splenic leiomyoma is a rare benign smooth muscle tumor of the spleen, most commonly reported in immunocompromised patients, including those with post-renal transplantation status, AIDS, or genetic disorders such as ataxia-telangiectasia [19,20]. The pathogenesis in these cases is thought to be associated with Epstein-Barr virus-related smooth muscle proliferation occurring in the context of an impaired immune system [19]. Most patients present with nonspecific abdominal pain, splenomegaly, cytopenias, or the tumor is found as an incidental lesion [19, 20]. Histologically, splenic leiomyomas resemble leiomyomas found elsewhere in the body, consisting of spindle-shaped smooth muscle cells arranged in interlacing fascicles. Immunohistochemistry shows positivity for smooth muscle actin and desmin, which helps distinguish them from other spindle cell neoplasms. Pediatric reports are exceedingly scarce. In a review of 30 pediatric cases with benign splenic lesions, leiomyoma was identified in only a single patient [16]. The first pediatric case described in the literature involved an 8-year-old boy with ataxia-telangiectasia [15]. Additionally, a case was reported involving an 18-year-old girl with an incidentally discovered splenic leiomyoma, which occurred alongside a duodenal ulcer, without any signs of immune deficiency [12]. Such observations indicate that splenic leiomyomas can also occur sporadically in patients with intact immune systems. 

Our case, therefore, expands the spectrum of clinical contexts in which splenic leiomyoma may occur by documenting its presence in an immunocompetent child with EGE, although the association between EGE and splenic leiomyoma appears to be incidental and has not been mechanistically explored.

## Conclusions

Our case is unique because it occurred in a child with EGE, an inflammatory disorder not typically linked to smooth muscle tumors, and it has not been shown to increase the risk of smooth muscle tumors. Although the presentation may represent a coincidental finding, it raises the possibility that splenic leiomyoma could develop in immunocompetent patients with underlying inflammatory disorders. Reporting such cases is important, as it expands the clinical contexts in which splenic leiomyomas may arise and contributes to a better understanding of their pathogenesis and natural history. Further accumulation of cases is needed to clarify any potential associations between inflammatory diseases and rare smooth muscle tumors of the spleen.
